# The impact of tree crops and temperature on the timing of frugivorous bird migration

**DOI:** 10.1007/s00442-020-04726-5

**Published:** 2020-08-06

**Authors:** Anna-Maria Kanerva, Tatu Hokkanen, Aleksi Lehikoinen, Kai Norrdahl, Jukka Suhonen

**Affiliations:** 1 Department of Biology, University of Turku, 20014 Turku, Finland; 2 Natural resources/Forest management, Natural Resources Institute Finland, PO Box 2, 00791 Helsinki, Finland; 3 The Helsinki Lab of Ornithology, The Finnish Museum of Natural History, University of Helsinki, PO Box 17, 00014 Helsinki, Finland

**Keywords:** Interaction between birds and trees, Frugivorous birds, Granivorous birds, Migratory behaviour, Mutualism, Species interactions, Tree crop size

## Abstract

Migration has evolved to tackle temporal changes in availability of resources. Climate change has been shown to affect the migration dates of species, which raises the question of whether the variation in the timing of migration is climate or resource dependent? The relative importance of temperature and availability of food as drivers of migration behaviour during both spring and autumn seasons has been poorly studied. Here, we investigated these patterns in frugivorous and granivorous birds (hereafter frugivorous) that are assumed to postpone their autumn migration when there is plenty of food available, which may also advance upcoming spring migration. On the other hand, especially spring migration dates have been negatively connected with increasing temperatures. We tested whether the autumn and spring migration dates of eleven common frugivorous birds depended on the crop size of trees or ambient temperatures using 29 years of data in Finland. The increased crop sizes of trees delayed autumn migration dates; whereas, autumn temperature did not show a significant connection. We also observed a temporal trend towards later departure. Increasing temperature and crop sizes advanced spring arrival dates. Our results support the hypothesis that the timing of autumn migration in the frugivorous birds depends on the availability of food and is weakly connected with the variation in temperature. Importantly, crop size can have carry-over effects and affect the timing of spring arrival possibly because birds have overwintered closer to the breeding grounds after an abundant crop year.

## Introduction

Each year, a large number of animals conduct migration journeys due to temporal changes in resources (Dingle 1996). In the boreal zone, the seeds of several tree species are an important but fluctuating food resource for migrating and wintering birds (Haila et al. 1986; Newton 2008; Meller et al. 2016). The year-to-year variation in food availability affects the migration behaviour of some migratory species, such as frugivorous and granivorous (hereafter frugivorous) birds at northern latitudes (Haila et al. 1986; Fox et al. 2009; Lindén et al. 2011; Suhonen and Jokimäki 2015; Suhonen et al. 2017) as well as southern latitudes (Griffioen and Clarke 2002; Mackay and Gross 2019).

Due to year-to-year variation in food resources, frugivorous birds may adjust their autumn migration period according to crop size of trees (Jenni and Kery 2003; Newton 2008). Recent work has shown that crop sizes of four common tree species are synchronous in space and time, and this synchrony is partly linked with annual changes in weather conditions (Meller et al. 2016; Gallego Zamorano et al. 2018). As crop failure or mast in the most common tree species is synchronous across large spatial scales, food availability for various frugivorous species is also likely to be synchronous.

Several not mutually exclusively hypotheses have been proposed to explain why some individuals migrate while others do not. Some hypotheses focus on intraspecific differences in sex, body size, or dominance status (Ketterson and Nolan 1976; Gauthreaux 1982; Lehikoinen et al. 2017); while, others stress variation in environmental conditions, especially temperature and the availability of food (Haila et al. 1986; Newton 2008). On the other hand, a growing body of literature has shown that increasing temperatures due to climate change have advanced the timing of spring migration in several taxa (Jonzen et al. 2006); whereas, the responses during the autumn period are not as clear (Jenni and Kery 2003; Lehikoinen 2011). This suggests that temperature may play a smaller role in the autumn migration than in the spring migration. As far as we know, there have been no previous studies that would have analysed how food availability during the autumn migration period might affect the timing of spring arrival. Increased food availability is assumed to increase the proportion of resident individuals or delay autumn migration (Newton 2008), but the relative importance of food availability vs. temperature is poorly known.

In this study, we tested whether the adjustment of autumn migration dates to seed crop size is a general phenomenon among frugivorous birds and whether the crop size affects the timing of migration more than the prevailing temperature. We also tested whether the autumn crop size or the median migration date in autumn has carry-over effects on the timing of spring arrival. We quantified the seed crop of four tree species that provide the most exploited food source for overwintering frugivorous birds in Finland (Meller et al. 2016; Gallego Zamorano et al. 2018) and related the crop size to the median migration dates of eleven common frugivorous bird species. All bird species were short-distance migrants. We hypothesise that the autumn migration of frugivorous species is delayed during years with a high crop size of trees and early autumn migration would occur during years of crop failure. Furthermore, we hypothesise that autumn food availability has carry-over effects on the spring migration of these species. In years with high food abundance, delayed autumn migration may cause birds to winter closer to their breeding grounds and thus, they may arrive earlier in spring. In contrast, spring arrival should be later after years of crop failure as birds may have spent their winter further away. An alternative hypothesis is that weather conditions are more important drivers of migration dates (Vähätalo et al. 2004; Newton 2008). This would mean that colder autumn and warmer spring weather conditions would advance autumn and spring migration, respectively. The hypotheses are not mutually exclusive and both drivers may operate at the same time.

## Materials and methods

### Seed and berry crop data

We used yearly seed crop size from silver and downy birch (*Betula pendula* and *Betula pubescens*, combined as birch species *Betula* spp.) and Norway spruce (*Picea abies,* hereafter spruce) and fruit crop size of rowanberry (*Sorbus aucuparia*) in Finland (Gallego Zamorano et al. 2018). The crop estimates for spruce, silver and downy birch are based on the monitoring programme of the Natural Resources Institute Finland; the crop sizes of the trees have been estimated for tens of stands across the country since 1979 (detailed methodology in Gallego Zamorano et al. 2018). The crop sizes of rowanberry have been collected as a part of the Finnish winter bird monitoring since 1986, where observers estimate the initial rowanberry crop in early winter using the relative abundance scale of six categories from no berries to very abundant (Gallego Zamorano et al. 2018).

### Bird migratory data

The spring and autumn migration counts of migratory birds have been collected at the Hanko Bird Observatory (59°48′ N, 22°53′ E), Southwest Finland since 1979. Here, we used 29 years (1986–2014) of autumn median departure days of eleven common frugivorous bird species in Finland (Table 1). These species are mainly short-distance migrants that winter usually in western, central or southern Europe, or are partially migratory, i.e. some individuals over-winter in Finland. Spring arrival median days included 27 years of data (1987–2014). Data from the spring 1990 are missing due to lack of observation effort. The phenology of migratory bird species was monitored at Hanko using standardised migration counts (including four hours standardised migration observation from the sunrise and counts of staging birds) and trapping data (including standardised mist-netting sites) from 25 July to 5 November (Vähätalo et al. 2004; Lehikoinen 2011; Lehikoinen and Jaatinen 2012). The combined data of all observation activities including the number of trapped birds resulted in a daily bird count. Observation activity covered over 95% of observation days annually and there was no trend in observation phenology (Lehikoinen 2011). Complete spring arrival data were available for eight bird species during 28 years; in three species, the number of years was 17–26. The migration date was recorded as the yearly cumulative number of days from 1st January [day 1 is 1st January; for more details see (Vähätalo et al. 2004)].

**Table 1:** The mean autumn and spring migration dates (day 1 is 1st January) with minimum (min) and maximum (max) dates for eleven short-distance migratory species feeding mainly on fruits (F) or seeds (G), *n* refers to the number of years

Species		Food	Autumn			*n*	Spring			*n*
			Mean	Min	Max		Mean	Min	Max	
Waxwing	*Bombycilla garrulus*	F	289	275	301	29	79	61	115	16
Blackbird	*Turdus merula*	F	269	259	294	29	85	71	107	27
Fieldfare	*Turdus pilaris*	F	285	255	305	29	96	70	113	27
Redwing	*Turdus iliacus*	F	273	259	305	29	96	72	113	27
Mistle Thrush	*Turdus viscivorus*	F	272	263	289	29	89	75	108	27
Starling	*Sturnus vulgaris*	F	275	248	293	29	87	71	107	27
Chaffinch	*Fringilla coelebs*	G	252	232	271	29	98	86	114	27
Brambling	*Fringilla montifringilla*	G	266	254	284	29	101	84	116	27
Siskin	*Carduelis spinus*	G	249	212	272	29	95	64	115	27
Redpoll	*Carduelis flammea*	G	282	264	305	29	91	65	114	27
Bullfinch	*Pyrrhula pyrrhula*	F/G	284	269	305	29	77	61	100	23
Total			272	212	305		91	61	116	

### Weather data

We calculated the autumn and early spring temperatures from southern Finland between 660 and 690 latitudes in the Finnish uniform coordinate system (59°40′–62°10′ N, 21°30′–32°00′ E), using weather data provided by the Finnish Meteorological Institute (Venäläinen et al. 2005). We used the mean September temperature for autumn migration, except for the late migrating species Bohemian Waxwing *Bombycilla garrulus*, Eurasian Blackbird *Turdus merula*, Fieldfare *Turdus pilaris,* Redpoll *Carduelis flammea* and Northern Bullfinch *Pyrrhula pyrrhula* (Lehikoinen and Vähätalo 2000), where we used the mean temperature of September and October. In spring, we used the mean March temperature in the analyses to describe the severity of the early spring.

### Statistical analyses

At the scale of southern Finland, tree crop sizes show strong spatial autocorrelation (Gallego Zamorano et al. 2018). To generate an uncorrelated crop size of tree species, we extracted principal components from the yearly crop size of the tree species (birch, spruce and rowanberry; Table 2).

**Table 2:** Correlation of yearly variation (*n* = 29 years) in the crop size with the principal component score (PCA1) in three species of trees

Species of tree	PCA1
Birch	0.787
Spruce	0.891
Rowan berry	0.838
Variance explained (%)	70.5

We used linear mixed models (LMM) to test which factors explain the median migration days of species during autumn and spring separately. In the full model, the fixed explanatory variables were crop size, temperature (September/October for autumn and March for spring) and year to account for potential linear trends in the migration dates. As random effects, models had for each study species separate intercepts and slopes for the crop size, the temperature and the year. The crop size and the temperature values were normalized (mean zero, standard deviation 1) before the analyses.

Furthermore, to test the connection between the autumn and spring migration dates, we used LMM, where the spring arrival dates of species were explained by the autumn migration dates of the previous year and year as a continuous variable. The autumn migration dates were centred species specifically before the analyses to account for species having different autumn migration periods. Species was a random factor in the analyses to account for that species have different spring arrival periods.

LMMs were calculated using the add-on package lmer (package of lme4) (Bates et al. 2015) and package visreg for drawing figure (Breheny and Burchett 2017) in R version 3.4.1 (RCore 2018). Correlations and principal component analyses were performed using the IBM SPSS statistical package, version 23.

## Results

The autumn and spring migration dates of the bird species varied, as expected. Most of the short-distance migrants departured at the end of September or early October and they arrived at late March or early April (Table 1).

**Fig. 1:**
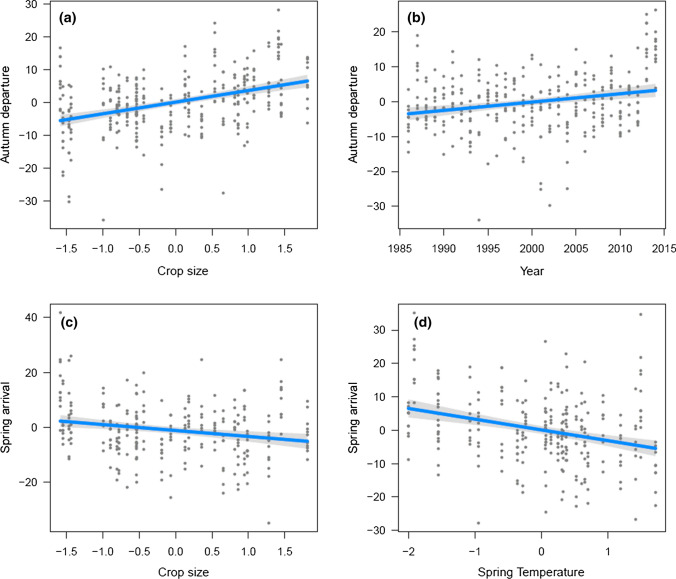
Median autumn migration dates of eleven frugivorous species in relation to **a** crop size of trees and **b** year, and median spring migration dates of the same species in relation to **c** crop size of trees and **d** early spring temperature in Finland

One axis (PCA1) accounted for 70.5% of the yearly variation in the crop size of trees (Table 2). Hereafter, we used PCA1 as an index of tree crop size. According to LMM analyses, increasing crop size delayed the autumn migration of birds, but there was also a temporal trend towards later departure (Fig. 1). Median autumn migration dates were not connected with mean autumn temperatures (Table 3).

**Table 3:** Coefficients of a linear mixed model (LMM) explaining the median autumn departure dates of eleven frugivorous bird species at Hanko peninsula

	*B* ± SE	*df*	*t* value	*P*
(Intercept)	272.44 ± 3.76	10.8	72.551	< 0.001
Year	0.23 ± 0.07	14.42	3.534	0.003
Temperature	0.49 ± 0.76	39.49	0.653	0.518
Crop size	3.55 ± 0.68	11.67	5.254	< 0.001

Temperature is the autumn temperature and Crop size is the PCA1 of crop sizes in birch trees, spruce and rowanberry

The spring arrival dates of birds were negatively associated with both the crop size and spring temperature (Fig. 1). Even though the coefficient of temperature was higher, it did not differ significantly from the coefficient of crop size, which suggests that both have the same magnitude of effect on the arrival dates of frugivorous species (Table 4).

**Table 4:** Coefficients of a linear mixed model (LMM) explaining the median spring arrival dates of 11 frugivorous bird species at Hanko peninsula

	*B* ± SE	*df*	*t* value	*P*
(Intercept)	91.35 ± 1.76	10.5	52.018	< 0.001
Year	− 0.12 ± 0.08	75.57	− 1.547	0.126
Temperature	− 3.47 ± 0.62	89.8	− 5.637	< 0.001
Crop size	− 2.74 ± 1.07	11.33	− 2.563	0.026

Temperature is the early spring temperature and Crop size is the PCA1 of crop sizes in birch trees, Norway spruce and rowanberry in the previous autumn

There was a tendency that spring arrival dates of species were negatively associated with the autumn migration dates of the previous year (*P* = 0.092), which suggests that later autumn migration can lead into earlier spring arrival (Table 5).

**Table 5:** Coefficients of a linear mixed model (LMM) explaining the median spring arrival dates of 11 frugivorous bird species at Hanko peninsula

	*B* ± SE	*df*	*t* value	*P*
(Intercept)	90.21 ± 2.16	10.8	41.778	< 0.001
Year	− 0.09 ± 0.08	269.5	− 1.10	0.272
Autumn	− 0.13 ± 0.08	268.9	− 1.69	0.092

Autumn refers to species specifically normalised median autumn migration dates

## Discussion

Our results support the hypothesis that the timing of both autumn and spring migration in the birds depends on the availability of food resources during autumn (Newton 2008). Furthermore, our results were in line with earlier findings suggesting that advancing spring arrival dates are linked with increasing temperatures (Lehikoinen et al. 2004, 2019; Gordo and Jose Sanz 2010; Lehikoinen and Sparks 2010). However, we did not find a connection between the timing of autumn migration and average temperatures. Our findings suggest that direct plastic responses in relation to food resource fluctuations are an important factor in explaining the inter-annual temporal variation in the migration of frugivorous bird species. The impact of food availability during autumn has also carry-over effects as increased crop size generally advanced the spring arrival in the next year. A plausible explanation for this observation is that species wintered closer to the breeding grounds during high crop years, which enables earlier spring arrival. Importantly, the impact of food availability during the previous autumn on arrival dates was of equal magnitude with the spring temperature.

There can be several reasons that are not mutually exclusive, why frugivorous birds delay their autumn migration. First, birds may try to minimise the costs of migration within the constraints set by food availability (Tyrväinen 1975; Robart et al. 2019). Second, wintering as close as possible may enable earlier spring migration to acquire better quality territory and breeding mate, but this idea needs more research. Third, delayed autumn migration probably decreases food competition in the southern wintering grounds and, thereby, increases the wintering survival, especially among young birds (Zuniga et al. 2017) that migrate earlier than adults (Lehikoinen et al. 2017). If delayed migration enhances wintering survival, rapid changes from fully migratory to partial migratory strategy may occur (Berthold et al. 1990).

Bird migration occurred earlier during years of warm early spring temperature as has been found in many earlier studies (Lehikoinen et al. 2004, 2019; Gordo and Jose Sanz 2010; Lehikoinen and Sparks 2010). In this study, all the species were early spring migrants that are frequently more irregular in their date of arrival than the late long-distance migrants, because the former are more likely to be delayed by poor weather (Elkins 1988). In particular, ground-feeding species such as thrushes, Starling (*Sturnus vulgaris*) and Chaffinch (*Fringilla coelebs*) arrived later during the cold springs. This may be linked with prolonged snow cover, as snow limit access to food.

In autumn, mean temperatures could not explain the timing of autumn migration but there was a temporal trend towards later departure. Delayed autumn migration dates are expected among short-distance migrants due to climate change (Jenni and Kery 2003; Lehikoinen and Jaatinen 2012). If the temporal trend was caused by warming climate, monthly mean temperature may be a too coarse variable to explain departure dates or the impact of climate can be non-linear. The temporal trend may also reflect lower energy needs due to warmer climate as well as a positive trend in the availability of food in the autumn, either through increased food production in late growing season, a reduced number of consumers, or due to changes in the availability of potential food sources.

Even though we could not find direct effect of temperature on autumn phenology, the temperature can indirectly affect the timing of migration. The crop sizes of the study tree species are found to be associated with temperatures with one or two year time-lag (Gallego Zamorano et al. 2018). Furthermore, the crop sizes of rowanberries have increased in Finland since 1980s, but the crop sizes of birches have decreased during the same time period. Production of spruce did not show any trend. The reason for these differing trends is not known, but especially species which are specialised on rowanberries could have more favourable feeding conditions and, thus, delay autumn migration.
